# Photodynamic and photothermal therapies for bacterial infection treatment

**DOI:** 10.1002/smo.20220010

**Published:** 2023-05-16

**Authors:** Heejeong Kim, You Rim Lee, Hyunsun Jeong, Jiah Lee, Xiaofeng Wu, Haidong Li, Juyoung Yoon

**Affiliations:** ^1^ Department of Chemistry and Nanoscience Ewha Womans University Seoul Korea; ^2^ School of Bioengineering Dalian University of Technology Dalian China

**Keywords:** AIEgens, antibacterial phototherapy, photodynamic therapy, photothermal therapy, phthalocyanine

## Abstract

Bacteria can cause numerous infectious diseases and has been a major threat to human humans. Although antibiotics have partially succeeded in treating bacteria, owing to antibiotic abuse, the emergence of multidrug‐resistant (MDR) bacteria has drastically diminished their potency. Since the invention of laser, the combination of light and photosensitizers, photodynamic therapy (PDT), has become an effective noninvasive treatment along with photothermal therapy (PTT), in which heat is generated by nonradiative relaxation. Antimicrobial PDT and PTT are emerging as effective treatments for bacterial infection, particularly against MDR bacteria. This mini review covers the recent progresses in PDT and PTT for bacterial treatment.

## Graphical abstract

This mini review covers the recent progresses in photodynamic therapy and photothermal therapy for bacterial treatment.

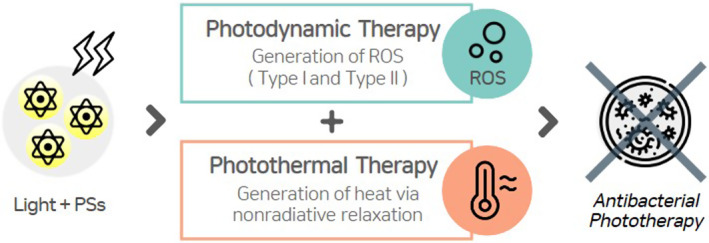

## 1 INTRODUCTION

Almost 3000 years ago, psoralens were utilized with light for treating vitiligo in Egypt and India.^[1]^ In 1903, the Nobel Prize in Physiology or Medicine was awarded to Niels Finsen, who was recognized as the father of modern phototherapy.^[2]^ However, further development of photodynamic therapy (PDT) became possible only in the 1960s with the discovery of lasers. With the advent of lasers, the combination of light and photosensitizers (PSs) became an effective noninvasive treatment for tumors and bacterial infections. Ideally, nontoxic PSs can generate reactive oxygen species (ROS) in the presence of light to provide an excellent therapeutic effect on tumors^[3]^ and bacterial infections^[4]^ as well as a high therapeutic precision and only a few side effects. PSs follow two different PDT mechanisms to generate ROS in the presence of light. In the type II process, molecular oxygen is converted to singlet oxygen (^1^O_2_), whereas the type I mechanism is a radical process, which produces superoxide radicals (O_2_^•−^), hydroxyl radicals (OH•), and so on.^[5]^ Conversely, photothermal therapy (PTT) involves heat generation via nonradiative relaxation.^[6]^ Because PTT uses heat to kill tumor cells or to treat bacterial infections, the use of oxygen in PDT can be avoided. In addition, photoacoustic imaging is possible for this process.^[7]^

Bacteria is the source of numerous infectious diseases and is thus detrimental to human health.^[8]^ Infectious bacterial diseases can be partially treated by antibiotics although the unsupervised and rampant use of antibiotics have resulted in the emergence of multidrug‐resistant (MDR) bacteria, which has drastically diminished the potency of antibiotics.^[9]^ According to a recent report, 15% of COVID‐19 patients suffer from secondary bacterial infections, indicating that patients with weak immune systems are at risk of developing secondary bacterial infections.^[10]^

Understanding the difference between the membranes of gram‐negative and gram‐positive bacteria is also essential for designing a PS selective to gram‐positive bacteria or gram‐negative bacteria. The penetrability of PS into gram‐negative bacteria is lower than that into gram‐positive bacteria because of the complex membrane of the gram‐negative bacteria.^[11]^

Recently, antibacterial PDT and PTT, which show antibacterial effects depending on the PDT or PTT effect of PSs, have been actively studied.[^12^, ^13^] The recently reported potential application areas of PDT, PTT, and the ROS and heat produced by PSs are emerging as new strategies for antibacterial drug design and synergistic chemotherapy.^[3]^ Despite the various advantages of phototherapy that have been studied so far, low solubility of phototherapy agents in water, which induces poor light absorption and biocompatibility, must be solved to move on to clinical trials. In addition, low phototherapeutic efficiency due to the short diffusion distance of ROS should also be handled.^[4]^ In this mini review, we cover the recent advancements in PDT and PTT for bacterial infection treatment. The first part covers phthalocyanine‐ or porphyrin‐based PSs, and the second part introduces aggregation‐induced emission luminogens (AIEgens)‐based PSs.

## 2 PDT AND PTT FOR BACTERIA

### 2.1 Phthalocyanine‐ or porphyrin‐based PSs

The Q‐band of phthalocyanine at around 670–850 nm is attributed to its efficient π–π* transition; this efficient transition is an advantage of phthalocyanine over porphyrin. In addition, due to its planar structures, phthalocyanine or porphyrin is easy to form a supramolecular self‐assembled nanoparticle, so the activity of PDT and PTT effects can be moderately controlled. Accordingly, significant efforts have been devoted to the development of phthalocyanine‐based PSs in recent years, and studies on the type I PDT agent design using planar moiety have been actively published.^[14]^

**FIGURE 1:**
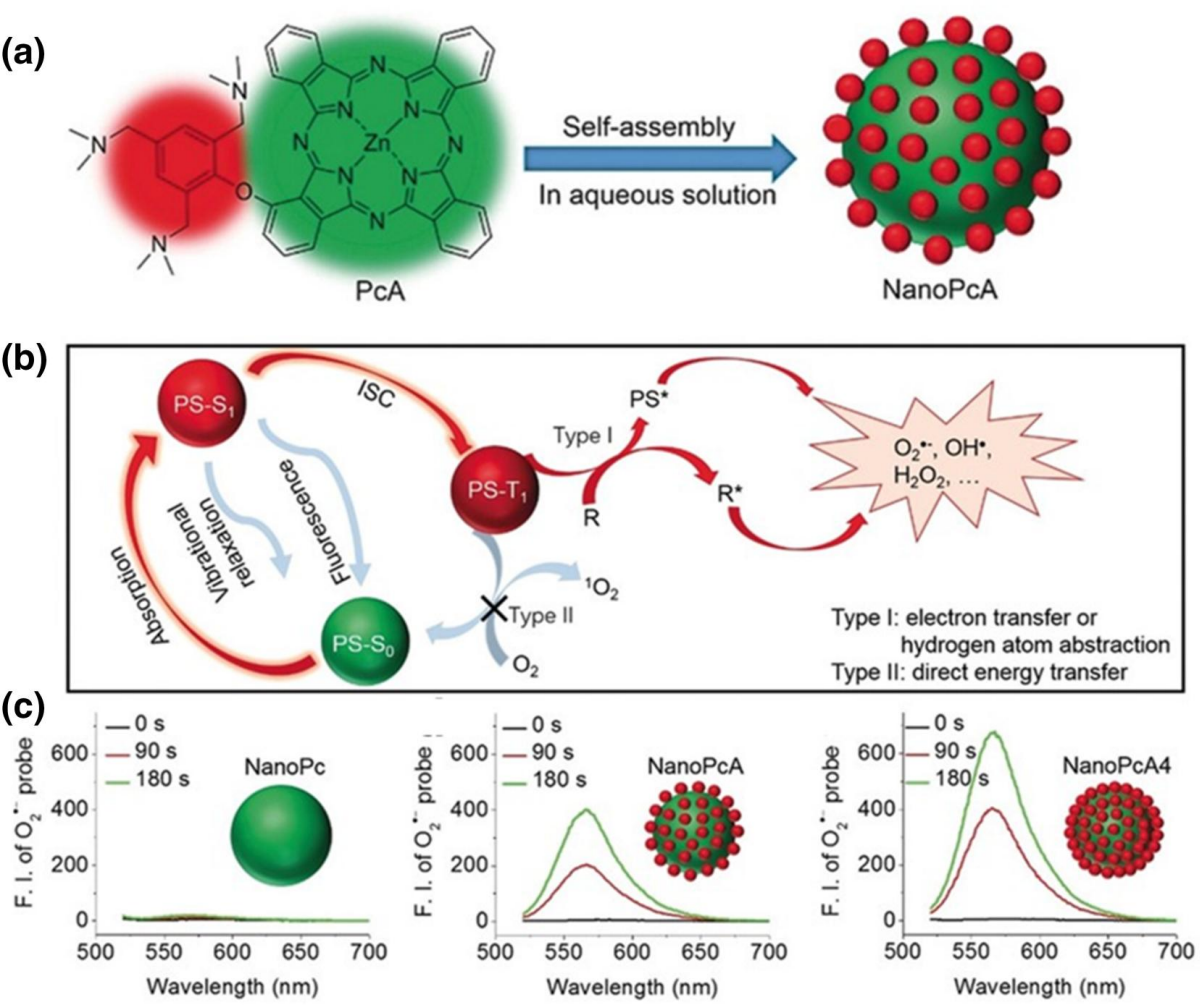
(a) Chemical structure of the PcA and schematic illustration of its nanostructured self‐assembly. (b) Schematic illustration of the potential mechanism of O_2_^•−^ production by NanoPcA. (c) Comparison of O_2_^•−^generation by NanoPc, NanoPcA, and NanoPcA4 in aqueous solutions. Reproduced with permission from Ref. [15]. Copyright (2018) Wiley‐VCH.

Numerous PSs reportedly achieved PDT through the oxygen‐dependent type II mechanism (^1^O_2_). However, the type I mechanism remains relatively unexplored.^[5]^ In this study, we designed and prepared a self‐assembled nanodot (NanoPcA; Pc represents phthalocyanine) in water using zinc (II) phthalocyanine substituted by 2,4,6‐tris‐(N,N‐dimethylaminomethyl)phenoxy (Figure 1a).^[15]^ Due to the presence of amino groups, the electron‐rich surface of NanoPcA suppressed the fluorescence emission and vibrational relaxation, which contributed to the formation of a triplet excited intersystem crossing (ISC) state and promoted ROS production via the type I mechanism (Figure 1b). In addition, as the number of amino groups on the surface of the nanodots increased, the yield of O_2_^•−^ also increased. This result demonstrated the crucial role of the electron‐rich surface of the nanoparticles in producing the ROS via type I photoreactions (Figure 1c). The NanoPcA was subsequently used for an antimicrobial type I PDT, which exhibited an excellent antibacterial efficacy for common and antibiotic‐resistant bacterial strains, such as spectrum beta‐lactamase *Escherichia coli* (ESBL *E. coli*) and methicillin‐resistant *Staphylococcus aureus* (MR *S. aureus*).

**FIGURE 2:**
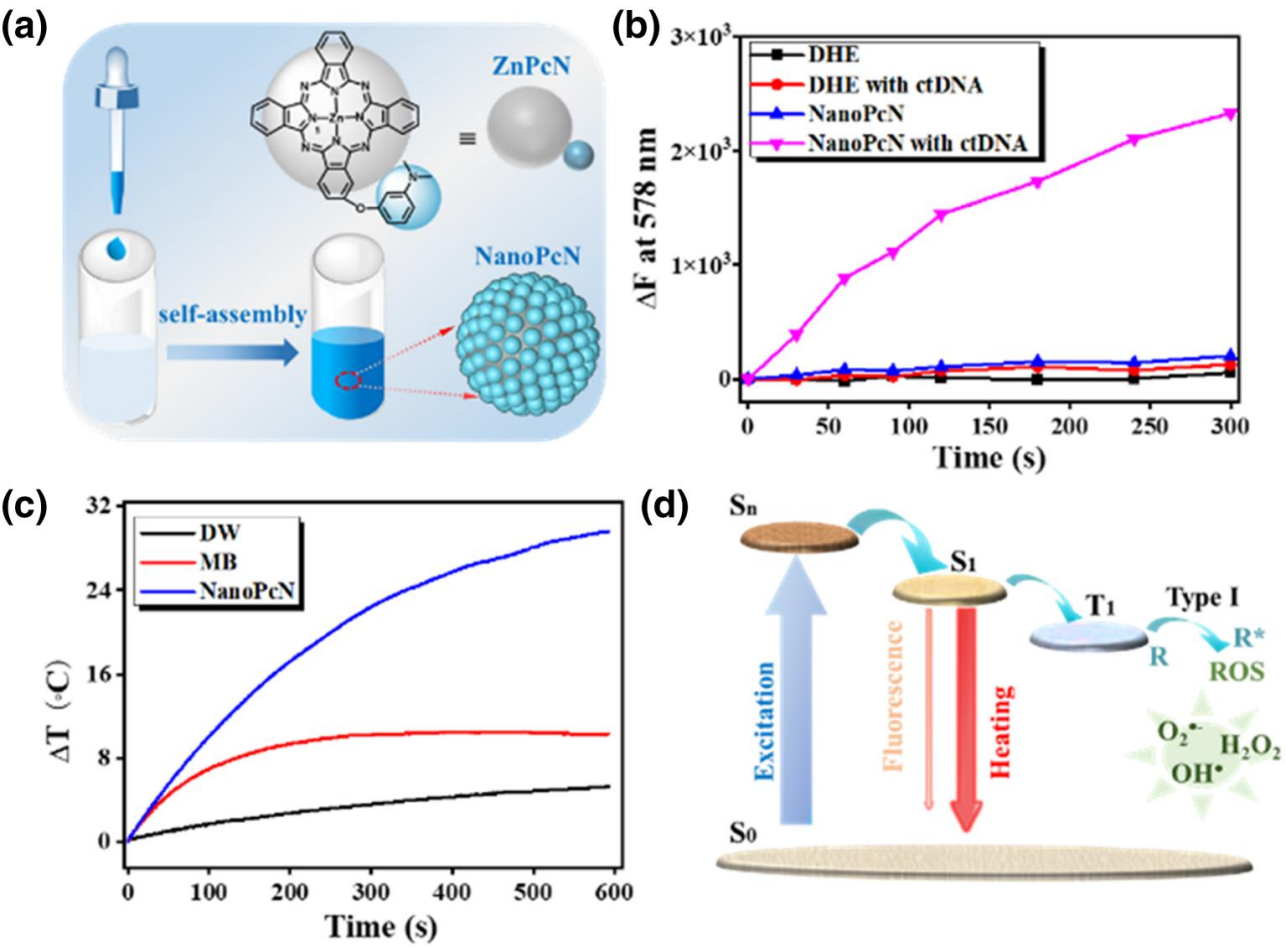
(a) Chemical structure of PcN and the structure of its self‐assembly NanoPcN. (b) O_2_^•−^ generation ability and (c) dynamic NanoPcN temperature after laser irradiation. (d) The potential photophysically activated NanoPcN process. Reproduced with permission from Ref. [17]. Copyright (2022) American Chemical Society.

Utilizing only a single treatment method may require higher doses and longer treatment periods to achieve the desired recovery. Dual or multimodal combination therapies frequently require the combination of multiple chemicals, resulting in cumbersome synthetic steps and poor biocompatibility.^[16]^ By adding a 3‐(dimethylamino) phenoxy group to zinc (II) phthalocyanine, we obtained PcN, which can undergo supramolecular self‐assembly to form NanoPcN in water (Figure 2a).^[17]^ The NanoPcN exhibited an excellent type I photodynamic activity and photothermal properties, which can be attributed to its amino group (Figure 2b,c). The lone pair of electrons on the amino group causes the photoinduced electron transfer (PET) effect of the phthalocyanine π system. As a result of this PET effect, the fluorescence characteristics of phthalocyanine are inhibited; additionally, it increases the likelihood of the generation of ROS and heat by NanoPcN via ISC and vibration relaxation, respectively (Figure 2d). Applying a “one‐for‐two” strategy inhibits the growth of both common and antibiotic‐resistant bacterial strains.

**FIGURE 3:**
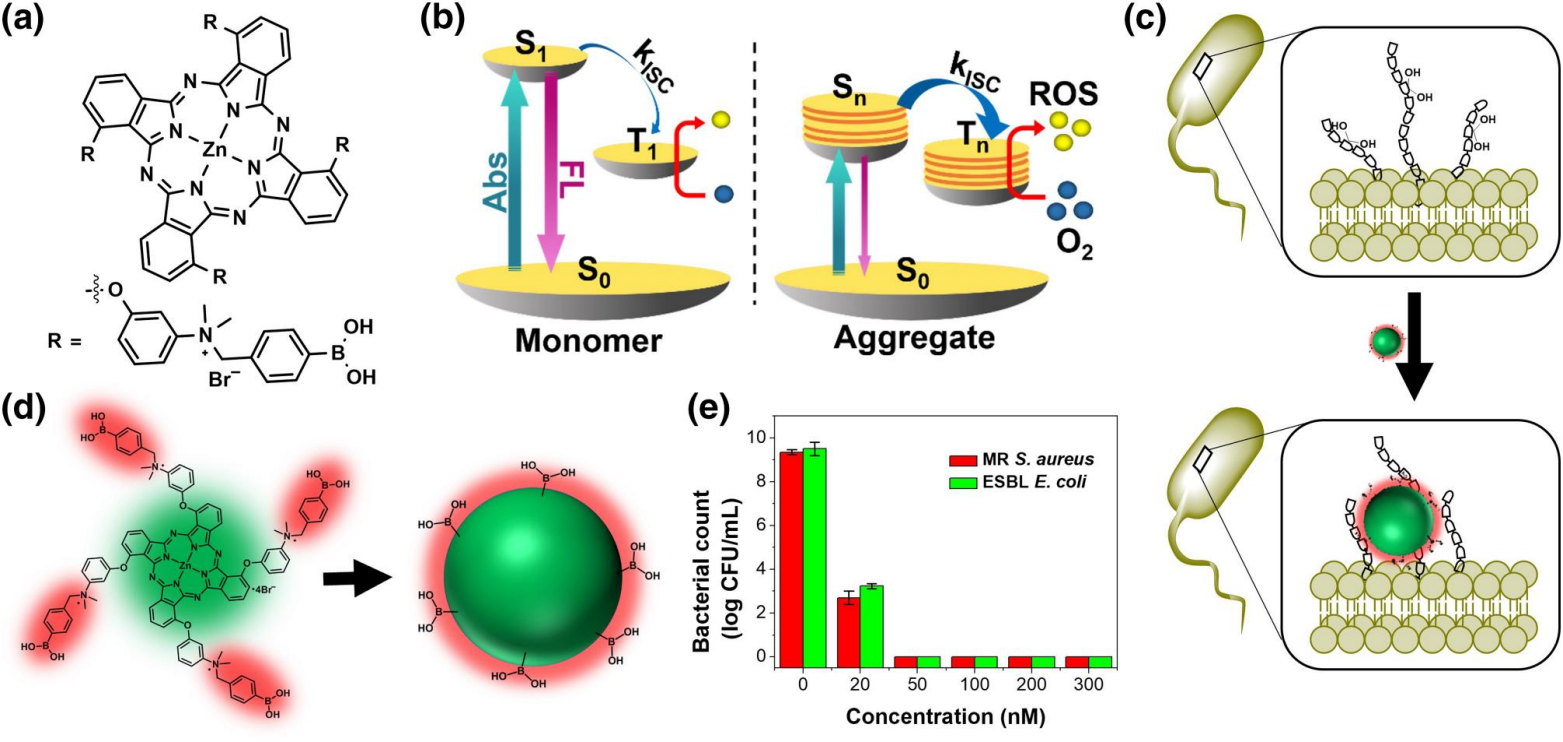
(a) Molecule structure of PcC4. (b) Enhanced ISC process of the aggregated state. (c) Schematic illustration of the binding of PcC4‐NA to the bacterial outer membrane. (d) Schematic depiction of the self‐assembled nanostructure formation. (e) Photoinactivation of MR *Staphylococcus aureus* and ESBL *Escherichia coli* in the presence of PcN4‐NA and under laser irradiation. Reproduced from Ref. [18] with permission from The Royal Society of Chemistry.

Transfer efficiency is the most important aspect of PS design to ensure an effective PDT. To enhance the tumor‐targeted delivery, quaternary ammonium and boronic acid (BA) were incorporated into zinc(II) phthalocyanine in order to improve its hydrophilicity and binding to the bacterial outer membrane. Interestingly, as the ratio of water to solvent increases, the amount of ROS produced by PcN4‐BA gradually increases due to the active ISC in the aggregated molecules (Figure 3).^[18]^ Similar to the in vitro experimental results, the number of active oxygen species generated by PcN4‐BA is significantly higher than that generated by several control groups without BA and methylene blue (MB), indicating that PcN4‐BA has a higher thermodynamic index. In addition, PcN4‐BA is not toxic to mammalian cells, suggesting that the incorporation of BA into the fluorophore could result in a high photodynamic antibacterial activity, as PcN4‐BA selectively binds to the bacterial outer membrane.

**FIGURE 4:**
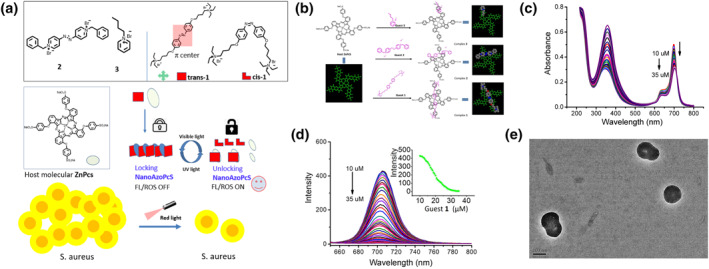
(a) Schematic illustration of the self‐assembly of **Azos**–**PS** nanoparticles. (b) Chemical structure of the **Azos**–**PS** host–guest systems. (c) Changes in the absorption spectra of **Azos**–**PS** host–guest systems. (d) Changes in the fluorescence spectra of the **Azos**–**PS** host–guest systems. (e) Transmission electron microscopy image of the **Azos**–**PS** host–guest systems. Reproduced from Ref. [19] with permission from The Royal Society of Chemistry.

Zinc phthalocyanine has many advantages as a PS in PDT because of its long absorption wavelength. Nevertheless, if the photodynamic effects of phthalocyanine can be controlled, then more effective and tunable photodynamic drugs can be manufactured. We synthesized a handful of new azobenzenes (**Azos**) and used them to regulate the self‐assembly behavior of phthalocyanine (i.e., the **PS**) (Figure 4a).^[19]^ Because of electrostatic attraction and π–π stacking interactions, Azos and phthalocyanine molecules form stable host–guest assemblies (Figure 4b). The assembly of Azos with the PS alters the absorption and fluorescence emission properties of the PS molecules (Figure 4c,d). In addition, electron microscopy results reveal that Azos and phthalocyanine molecules assemble into nanoparticles at high concentrations and that PDT can control the bacteria‐killing ability of the nanoparticles (Figure 4e).

**FIGURE 5:**
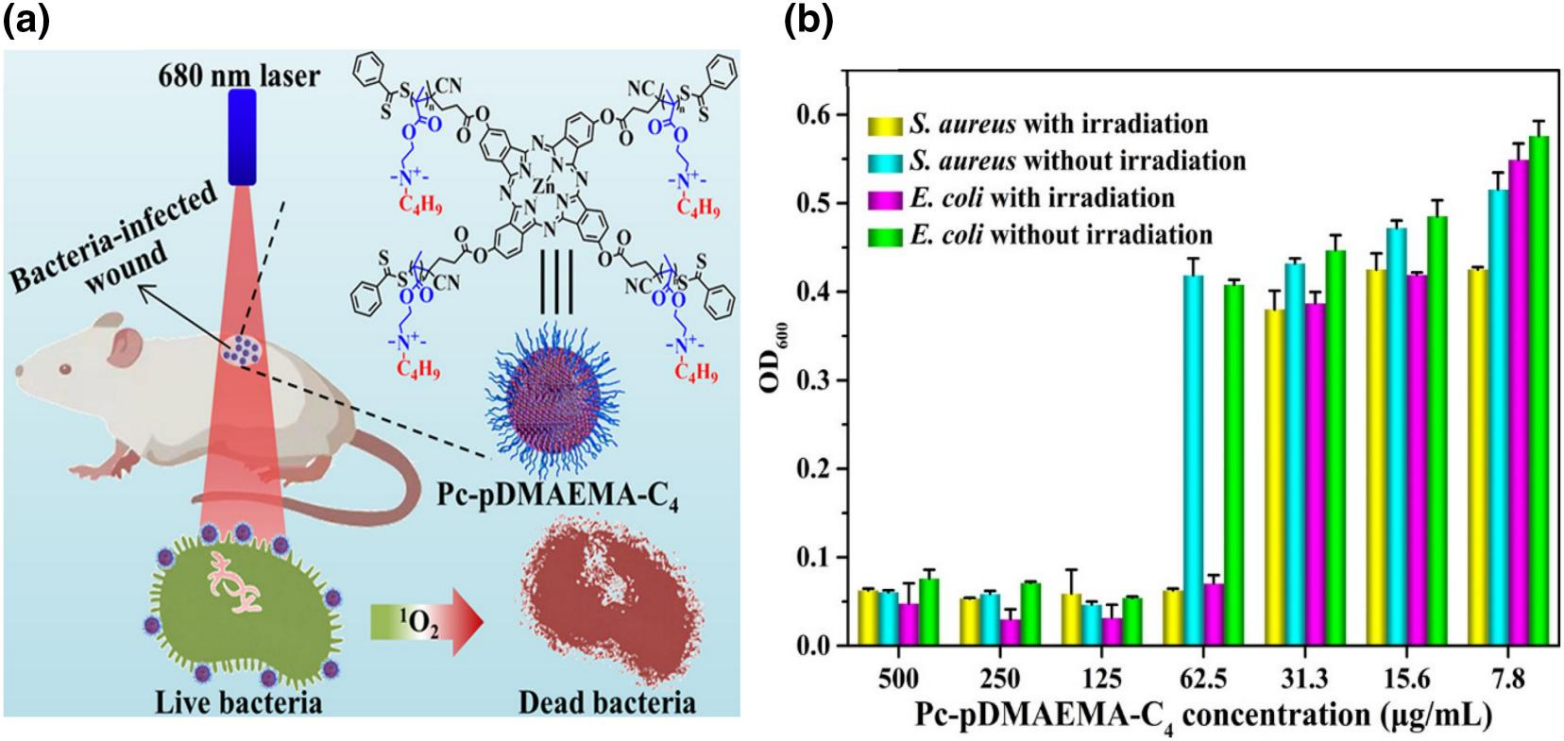
(a) Molecular structure of Pc‐pDMAEMA‐C4 and the schematic illustration of antibacterial PDT. (b) Bacterial growth inhibition using various concentrations of Pc‐pDMAEMA‐C4 with and without 680 nm light irradiation. Reproduced from Ref. [20]. Copyright (2022), with permission from Elsevier.

PDT is a growing area for antibacterial treatment, and phthalocyanine is a well‐reported PS because of its strong light absorption in the range of 650–800 nm, which leads to the generation of singlet oxygen (^1^O_2_). However, the poor solubility of phthalocyanine and lowered PDT efficiency are the major issues that need to be resolved. A phthalocyanine‐based cationic polymer Pc‐pDMAEMA‐C_4_ with an improved water solubility, a stronger photodynamic antibacterial effect, and a negligible cytotoxicity was synthesized by the Mei group (Figure 5a).^[20]^ Pc‐pDMAEMA‐C_4_ was prepared by the reversible addition fragmentation chain transfer polymerization using the DMAEMA monomer, followed by quaternization with 1‐bromobutane onto the tertiary amino group of the DMAEMA chain to form a cationic amino group. The hydrophilic p‐DMAEMA‐C_4_ chains and hydrophobic phthalocyanine self‐assemble to a polymeric nanomicelle in an aqueous environment and thus show enhanced water solubility and dispersibility. The cationic polymer chain provides a positively charged surface, which induces interactions with the negatively charged bacterial membrane. Accordingly, Pc‐pDMAEMA‐C_4_ shows dual antibacterial effects through ^1^O_2_ generation and the cationic polymer chain. The study of ^1^O_2_ generation with DMA trapping reagents illustrated that Pc‐pDMAEMA‐C_4_ generated ^1^O_2_ upon the irradiation of 680 nm light, and this caused higher bacterial cytoplasm leakage than without light treatment. Further in vitro experiments also revealed that only half the concentration of Pc‐pDMAEMA‐C_4_ was needed to effectively inhibit bacterial growth under the light‐irradiated condition than without the light‐irradiated condition (Figure 1b). In vivo experiments on bacterial infected rat also demonstrated rapid healing upon treatment with Pc‐pDMAEMA‐C_4_ and 10 min of light irradiation (wavelength: 680 nm). Thus, the cationic Pc‐pDMAEMA‐C_4_ promotes adsorption on the bacterial membrane and serves as an effective PS for antibacterial PDT.

**FIGURE 6:**
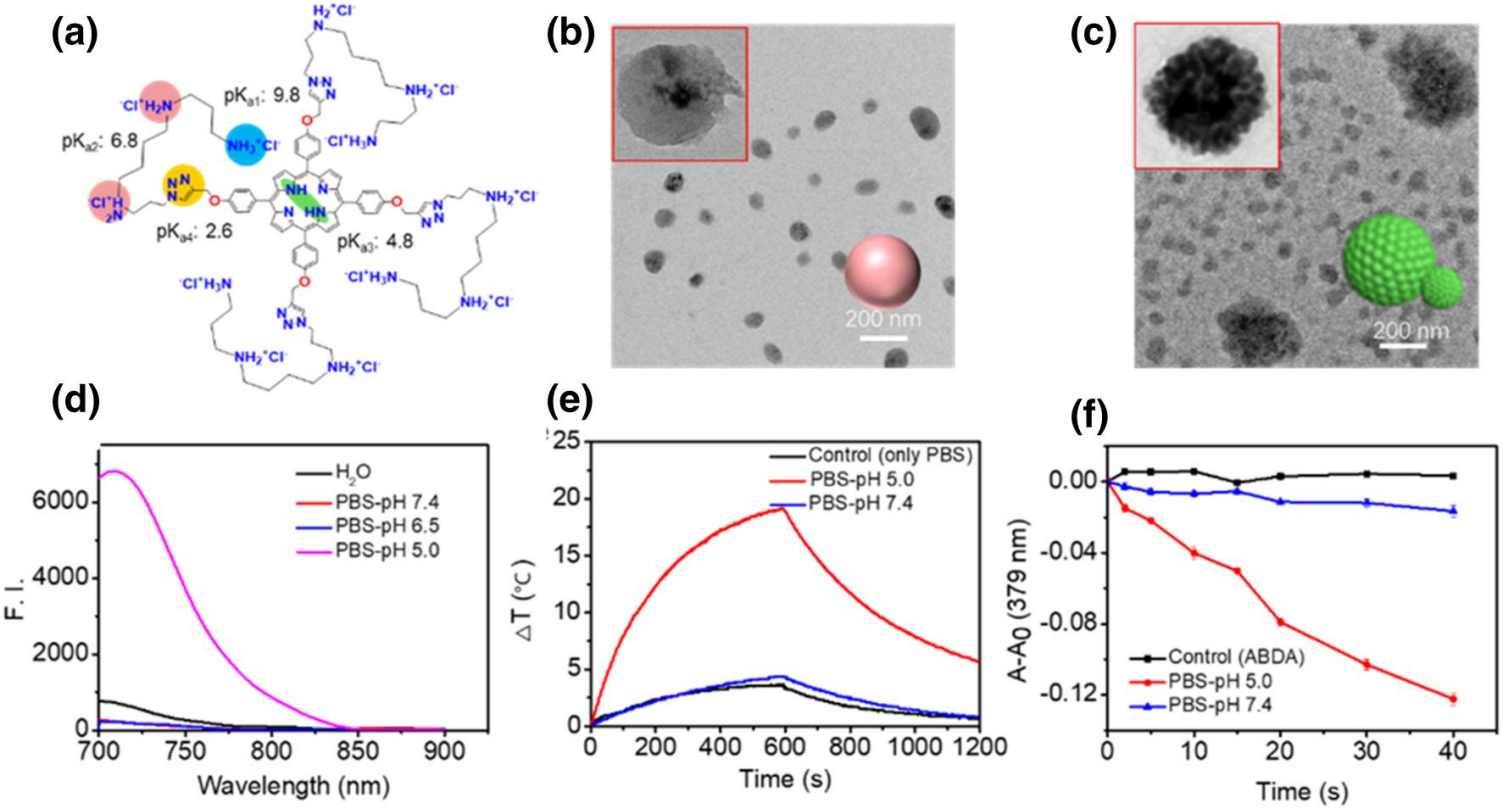
(a) Chemical structure of the water‐soluble porphyrin derivatives (PN3). Transmission electron microscopy images of PN3 in (b) PBS (pH 7.4) and (c) PBS (pH 5.0). (d) Fluorescence spectra, (e) photothermal performance, and (f) singlet oxygen generation by PN3 in different solutions. Reproduced with permission from Ref. [22]. Copyright (2022) Wiley‐VCH.

The effectiveness of the antibiotic treatment is inhibited by the nature of the bacterial resistance. Frequently, bacterial communities are surrounded by self‐produced extracellular polymer‐based biofilms that protect the bacteria.^[21]^ Additionally, the acidic and anaerobic environment of the biofilms reduces the effectiveness of antibiotics. Therefore, porphyrin derivatives (PN3) were synthesized to utilize the acidic environments. PN3 is water‐soluble and self‐assembles into a pH‐sensitive nanoporphyrin (PN3‐NP) that can be used for near‐infrared (NIR) fluorescence imaging and photoablation of biofilms (Figure 5a).^[22]^ The conjugate of spermine molecules with the porphyrin macrocycle provides additional kinetic control sites that allow PN3 to self‐assemble into stable nanoparticles (PN3‐NPs) in a physiological environment (Figure 6b). This process enables an efficient penetration of PN3‐NPs into the biofilms because of their favorable size. After penetrating into the biofilm, the acidic environment induces reassembly of the PN3‐NPs substructure under the influence of various weak interaction forces. Thus, the stable nanoparticles are converted into dot‐like nanospheres (Figure 6c). Notably, NIR fluorescence imaging and PTT and PDT effects are also simultaneously activated. The fluorescence maps of the biofilms can validate the activate NIR fluorescence imaging. In addition, crystal violet‐stained and scanning electron microscopy (SEM) images of the biofilms demonstrate that PN3‐NPs exude a strong photoablation effect on the biofilms under light irradiation due to the synergistic PTT and PDT effects (Figure 6e,f).

**FIGURE 7:**
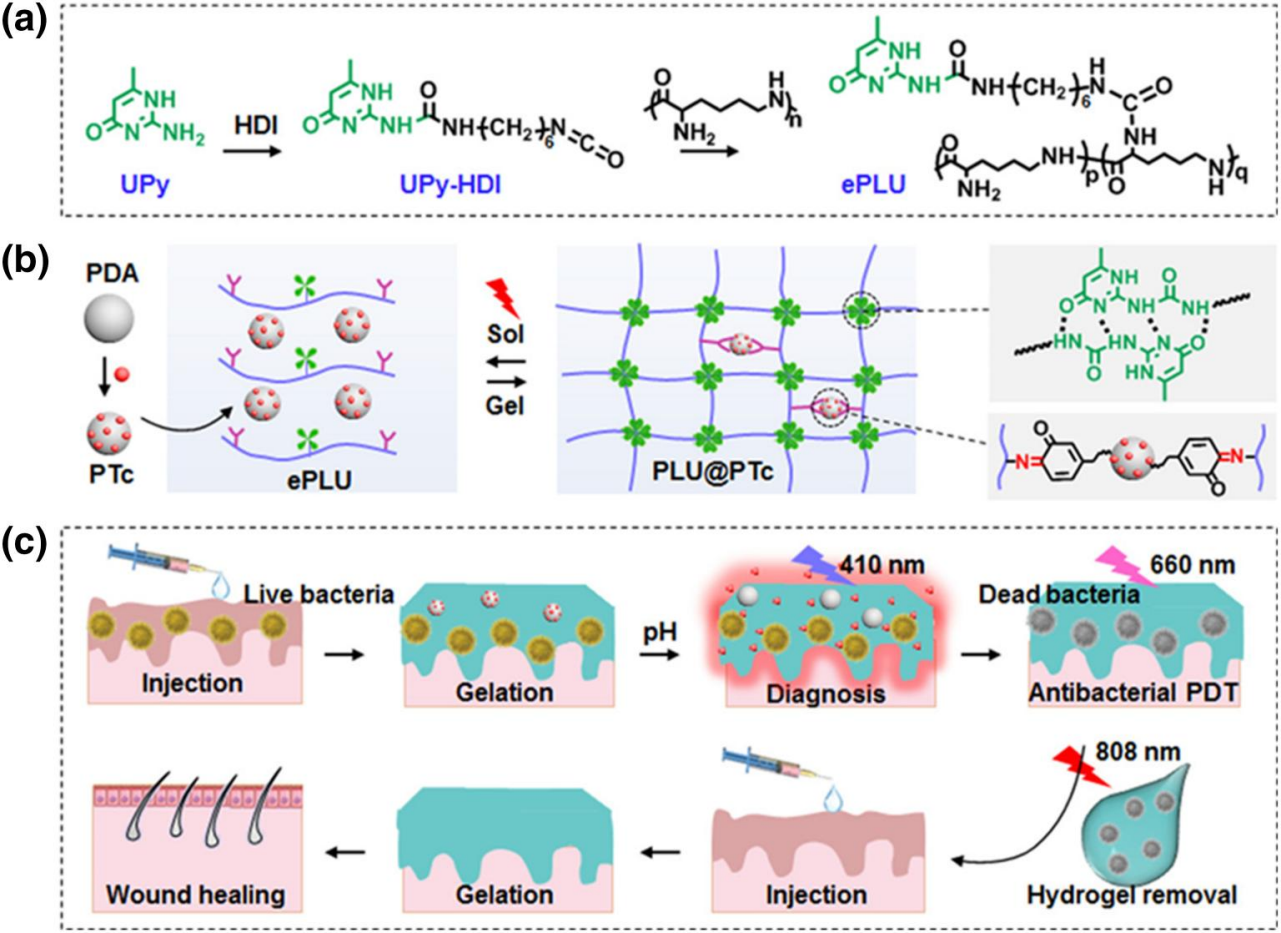
(a) Synthesis scheme of ePLU starting from UPy and ePL. (b) Illustration of cross‐linking system of PLU@PTc by hydrogen bonding and Schiff base linking. (c) Release of TCPP triggered by bacterial infection and wound healing process using wrapped bacterial debris with hydrogels. Reproduced from Ref. [24]. Copyright (2022) with permission from American Chemical Society.

The existing hydrogel dressing method cannot remove dead bacteria and bacterial debris, which can cause additional infections and causes pain and secondary wounds when removed. Recently, Li et al. overcame this weakness and devised a PLU@PTc hydrogel, which enables diagnosis, primary treatment, and secondary treatment.^[23]^ In this reported work, a hybrid hydrogel consisting of polydipamine (PDA) nanoparticles and ε‐polylysine (ePL), which is a typical antibiomicrobial peptide, was synthesized. Ureido‐pyrimidinone (UPy) was linked to hexamethylene diisocynate for conjugation with ePL; thus, the PLU hydrogels were cross‐linked by well‐known quadruple‐hydrogen bonds, which act as supramolecular self‐assembly moieties (Figure 7a). Tetrakis(4‐carboxyphenyl)‐porphyrin (TCPP) was further loaded via π−π stacking to form PTc nanoparticles as PSs. The PLU@PTs hydrogel can be synthesized through cross‐linking with PDA of the quinone group and ePLU of the amino group, and an in situ sol‐gel transformation is possible due to its Schiff base and hydrogen bonding. ePLU consisting of 20% UPy was selected in this study because of its bacteria capturing ability, injectability, and antibacterial effect. PDA nanoparticles can quench the fluorescence emission of TCPP and restore it through TCPP release in a low acidic environment of pH 5.5 due to bacterial infection, thereby allowing a real‐time bacterial infection diagnosis at 410 nm. In addition, only a negligible amount of TCPP is released at pH 7.4, thereby avoiding normal cell deaths and enabling a more accurate diagnosis and treatment. Under the illumination of 660 nm light, PDT can kill or catch bacteria, thereby overcoming the short lifespan of ROS, which is a conventional PDT disadvantage. After the treatment, the dead bacteria and their debris are wrapped within the PLU@PTc hydrogels due to the photothermal inversion capability of PDA. Further, the fluidity of hygrodels can be increased because of large temperature changes and sol‐gel transformation of the PLU@PTc under NIR irradiation (wavelength: 808 nm) (Figure 7).

### 2.2 AIEgens‐based PSs

Since Tang's pioneering work on aggregation‐induced emission (AIE) in 2001,^[25]^ significant efforts have been devoted to developing various AIEgens to prevent the aggregation‐caused quenching effect.^[26]^ A remarkable number of studies on AIE‐based platforms have been reported on the antibacterial efficacy of AIEgens because it can not only solve the conventional antibiotic resistance but also introduce various functional components.[^27^, ^28^] However, herein, we will cover only selected examples from our group and those from others.

**FIGURE 8:**
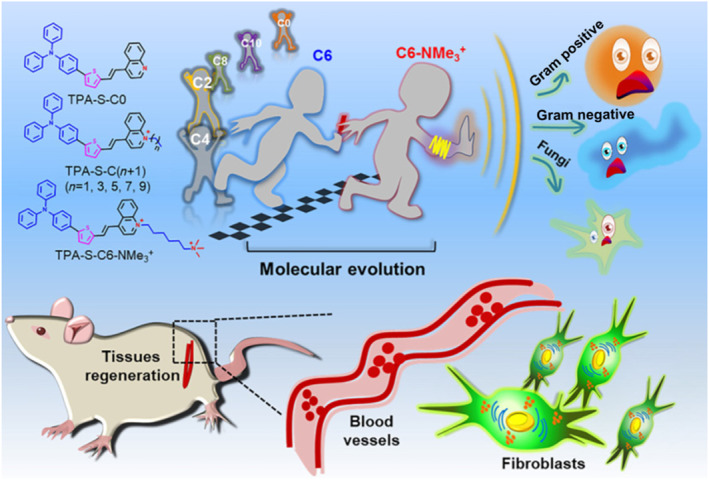
Design strategy and schematic illustration of the NIR‐smart AIEgen, **TPA‐S‐C6‐NMe**_**3**_^**+**^, for the inactivation of bacteria and fungi. Reproduced with permission from Ref. [29]. Copyright (2021), Chinese Chemical Society. AIEgen, aggregation‐induced emission luminogen; NIR, near‐infrared.

Infections caused by bacteria and fungi significantly impact global public health. In this study, we employed the concept of molecular relay and successfully developed an NIR‐smart AIEgen **TPA‐S‐C6‐NMe**_**3**_^**+**^ for the synergistic inactivation of bacteria and fungi to obtain a potent broad‐spectrum antimicrobial agent.^[29]^ As shown in Figure 8, by varying the length of the alkyl chain at the end of **TPA‐S‐C0**, a series of molecules (**TPA‐S‐C2–C10**) with varying ROS production capacities were designed. In vitro experiments demonstrated that the **TPA‐S‐C6** AIEgen is effective against *S. aureus* and *E. coli* but is ineffective against ESBL *E. coli*. We added a second positive charge to **TPA‐S‐C6** and easily prepared **TPA‐S‐C6‐NMe**_**3**_^**+**^, whose antimicrobial pathogen activity was superior to that of the commercial PSs (including protoporphyrin IX, chlorin e6, and MB). The results of this study demonstrate the importance of molecular size, ROS yield, and positive charge in designing a potent antimicrobial agent.

**FIGURE 9:**
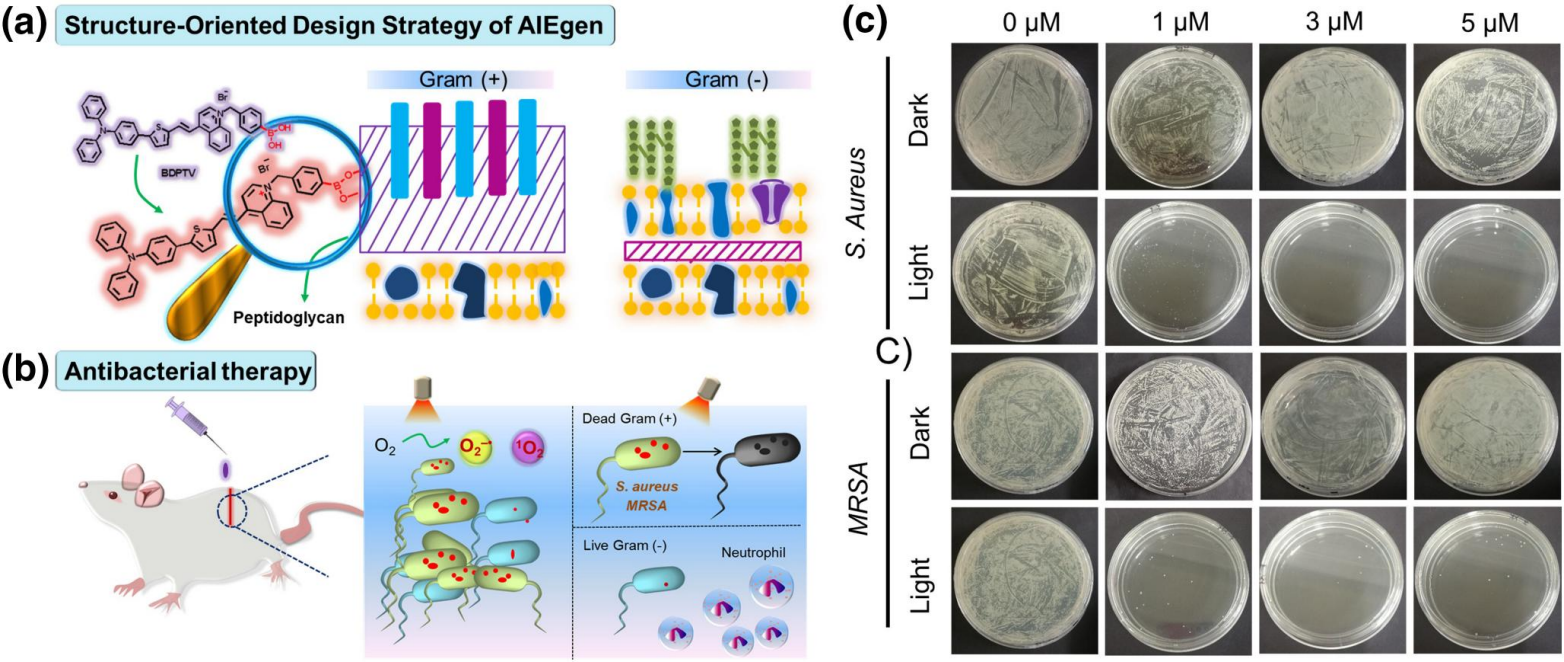
Schematic illustration of (a) SODS of the photo‐antimicrobial AIEgen BDPTV and (b) antibacterial therapy. (c) Plate photographs demonstrating the antibacterial effect of BDPTV on various bacteria when exposed to white light. Reproduced from Ref. [31]. Copyright (2022) with permission from Elsevier. AIEgen, aggregation‐induced emission luminogen; SODS, structure‐oriented design strategy.

Gram‐positive bacteria, which are common pathogens, cause serious yearly infections. However, to date, only a few studies have been reported on their selective killing, particularly for the drug‐resistant methicillin‐resistant *Staphylococcus aureus* (MRSA).^[30]^ The peptidoglycan layer of gram‐positive bacteria is thicker than that of the gram‐negative bacteria (Figure 9a); therefore, an organic AIEgen, that is, BDPTV, for antibacterial activity under light irradiation was designed using a bacterial structure‐oriented design strategy (SODS). In this SODS, a phenyboronic acid moiety was included in the BDPTV bonded to the peptidoglycan layer via covalent bonds.^[31]^ The phenylboronic acid moiety is linked to the molecule, so BDPTV can tightly attach to the bacterial cell wall in a confined space, and a positive therapeutic effect can be obtained (Figure 9b). In vitro experimental comparisons of gram‐positive and gram‐negative bacteria showed that BDPTV has excellent selectivity of gram‐positive bacteria. In this case, a positive bacteria (*S. Aureus*) and drug‐resistant positive bacteria (*MRSA*) were nearly eradicated by light irradiation, whereas the negative bacteria (*E. coli* and ESBL‐EC) were only negligible affected (Figure 9c). Comparative molecules without phenylboronic acid do not exhibit any bactericidal effect; this confirms the bacterial effect mechanism. In addition, the antibacterial effect of BDPTV is superior to that of the commercial PSs according to both in vitro and in vivo experimental results.

**FIGURE 10:**
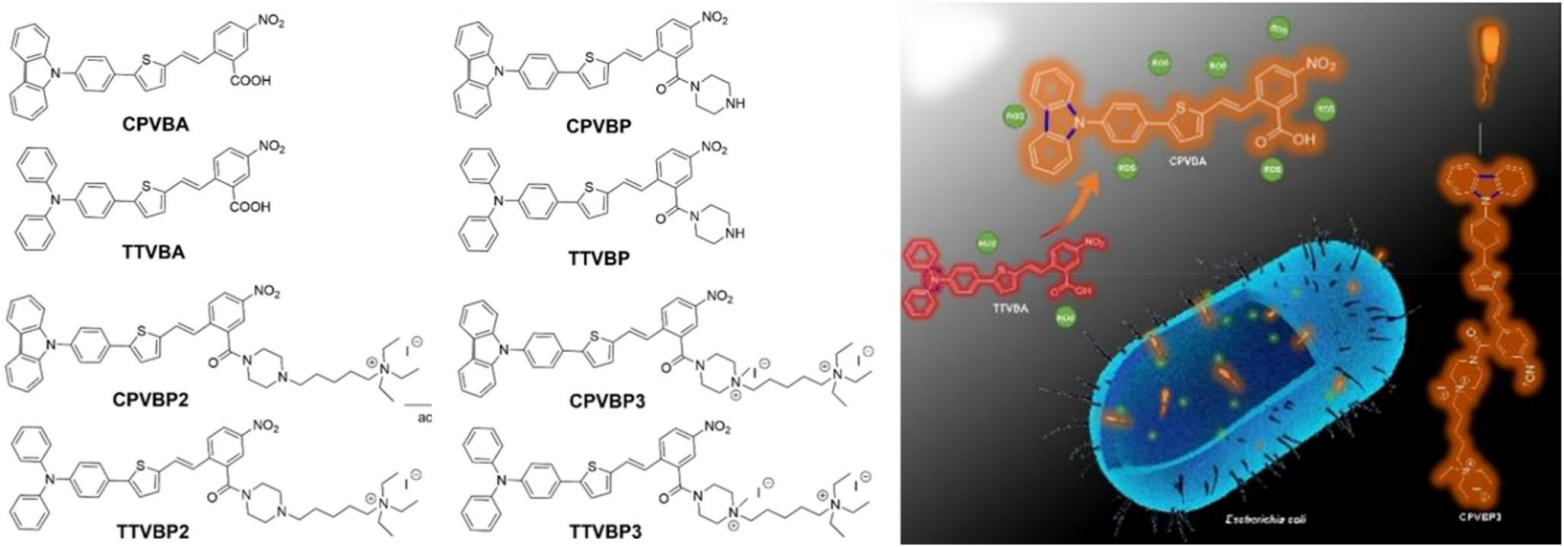
Molecular structure of the CPVBP and TTVBP derivatives. Reproduced from Ref. [32]. Copyright (2022) with permission from American Chemical Society.

Several strategies have been applied to design better AIEgens for antibacterial photodynamic therapy (aPDT), and one of them is positioning the AIEgens in a microbial environment to increase antibacterial efficacy by minimizing the intramolecular rotation of the covalent bonds. Chen's group utilized this concept and synthesized CPVBP and its derivatives using carbazole rings, which can completely prohibit the rotation of triphenyl amines (based on their previous study on TTVBP) (Figure 10).^[32]^ As a result, CPVBP can generate more active oxygen species while taking full advantage of the AIE phenomenon. In particular, time‐dependent density functional theory calculations show that CPVBP derivatives have a higher dEST than the corresponding TTVBP. This result indicates that limiting the nonradiative decay pathway can effectively increase the generation of ROS despite the reduced ISC effect. Based on the above in vitro results, the aPDT efficacy was tested using various bacterial groups. CPVBP2 and CPVBP3 showed the highest antibacterial ability for gram‐positive and CPVBP3 gram‐negative bacteria, respectively. To explain the difference in the antimicrobial effects according to the structure of bacteria, measurement of zeta potential and SEM imaging were performed. The corresponding measurement and imaging results proved that CPVBP3 can penetrate the gram‐negative bacteria more easily and can thus enhance the antibacterial action of the ROS. In conclusion, carbazole‐group‐based AIE‐PSs indicate that limiting the free molecular motion can actively promote ROS generation under light irradiation, and this process is advantageous for improving the molecular designs for PDT.

**FIGURE 11:**
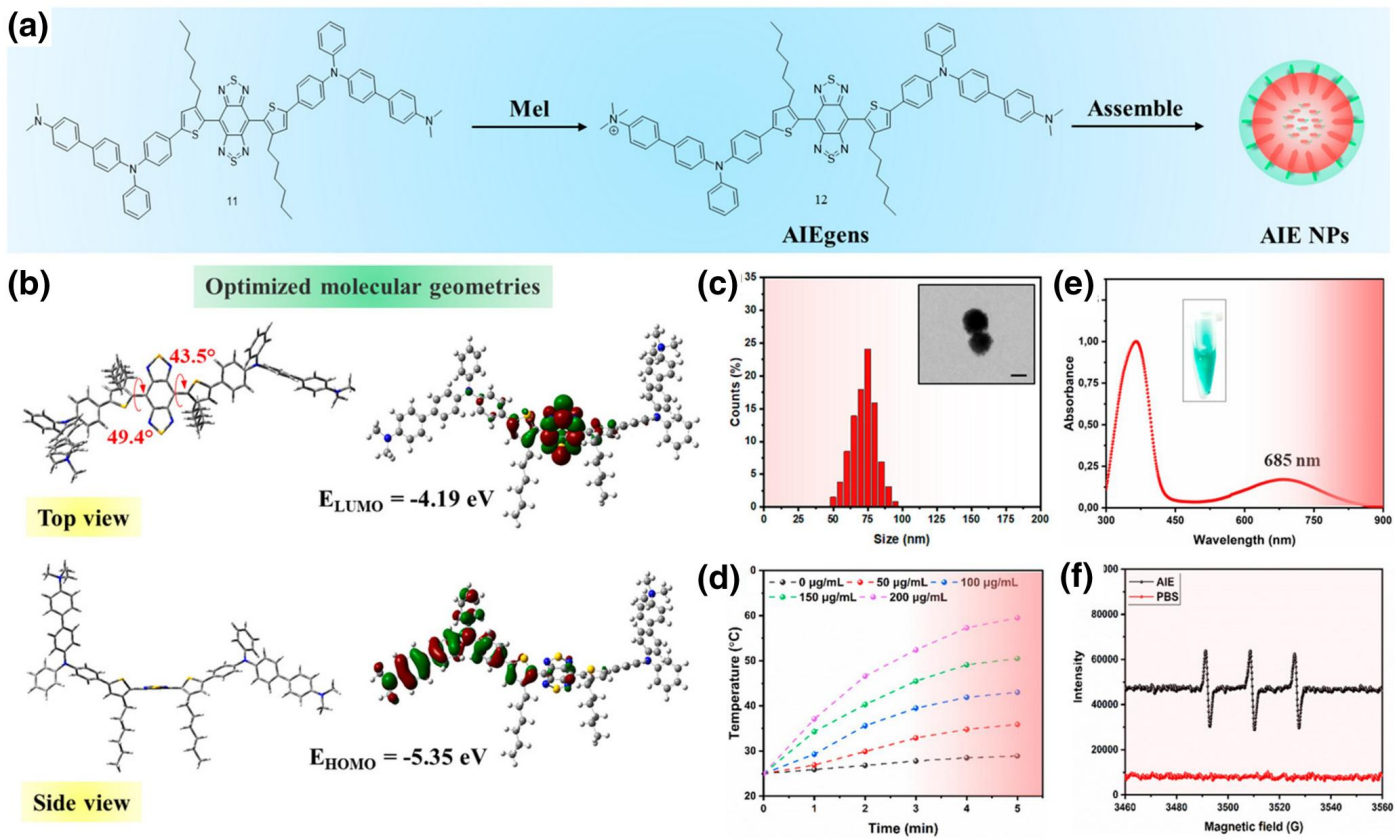
Synthesis of AIEgens (a) and formation of spherical AIE NPs (a, c). Least unoccupied molecular orbital and highest occupied molecular orbital (b) of the AIE NPs, which can strongly absorb NIR light (e) and photothermal effect (d). Singlet oxygen generation upon 808‐nm laser irradiation (f). Reproduced from Ref. [24]. Copyright (2022), with permission from American Chemical Society. AIEgens, aggregation‐induced emission luminogens; NIR, near‐infrared.

To date, PDT and PTT, which utilize PSs, have been mainly considered to solve the MDR bacteria ablation problem. Tang and coworkers synthesized AIEgens and reported that a positive quaternary amine group introduced to a highly twisted compound facilitates ROS generation and confers AIE properties.^[24]^ Spherical nanoparticles (AIE‐NPs) were generated by self‐assembly (Figure 11a), and the prepared AIE NP solution exhibited an increase in temperature and a high singlet‐oxygen level under an 808 nm laser (Figure 11). Only the bacteria groups treated with the AIE NPs under the irradiation of an 808 nm laser produced endogenous ROS. Particularly, the AIE NPs activated PTT/PDT under a high‐power laser (0.2 W/cm^2^) and showed a nearly 100% antibacterial effect. In vivo experiments were conducted on mice with skin wounds infected with *S. aureus*. After the anti‐infection treatment implemented through the AIE NP‐induced PTT/PDT, temperature enhancement and mass production of singlet oxygens were observed in the wound site. As expected, the AIE NPs combined with a high‐power laser displayed the best wound‐healing capacity and antibacterial ability.

**FIGURE 12:**
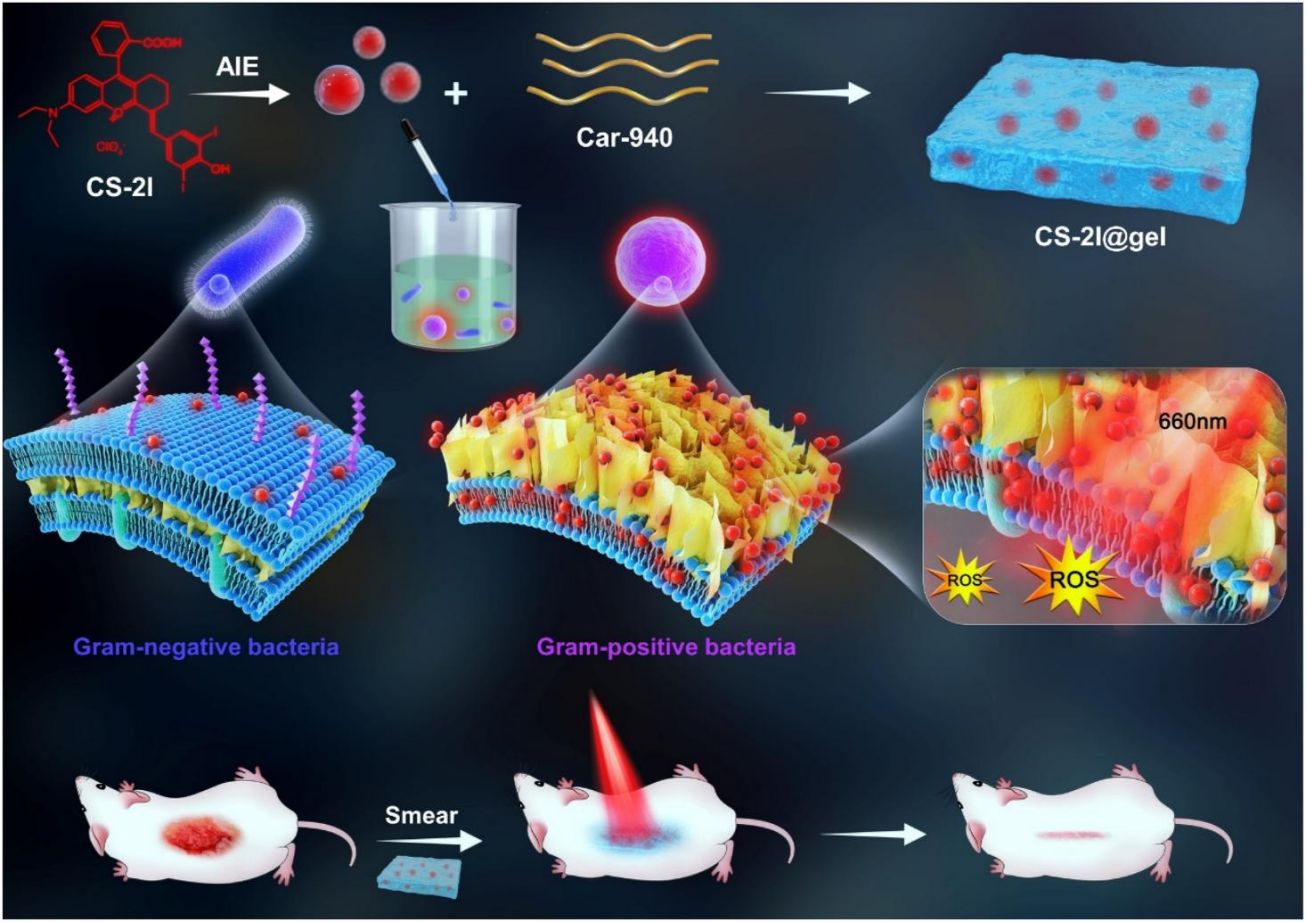
Chemical structure of the **CS‐2I** AIEgen and the application of **CS‐2I@gel** in the inactivation of bacteria via PDT. Reproduced with permission from Ref. [33]. Copyright (2022) Wiley‐VCH. AIEgen, aggregation‐induced emission luminogen; PDT, photodynamic therapy.

Recently, an AIE hydrogel **CS‐2I@gel** (Figure 12) was synthesized by combining **CS‐2I** (AIEgen) and Carbomer 940.^[33]^ Further, the first rhodamine‐based AIE PS, **CS‐2I**, was prepared by incorporating iodine atoms. The excellent photostability, NIR fluorescence emission, and ^1^O_2_ production capacity of CS‐2I make it suitable for selectively staining and killing gram‐positive bacteria (including MRSA) under photoirradiation. Due to the benefits of hydrogel biomaterials, **CS‐2I@gel** was successfully applied to in vivo anti‐MRSA activity and to the healing of wounds in mice.

### 2.3 Other PSs for antimicrobial PDT

**FIGURE 13:**
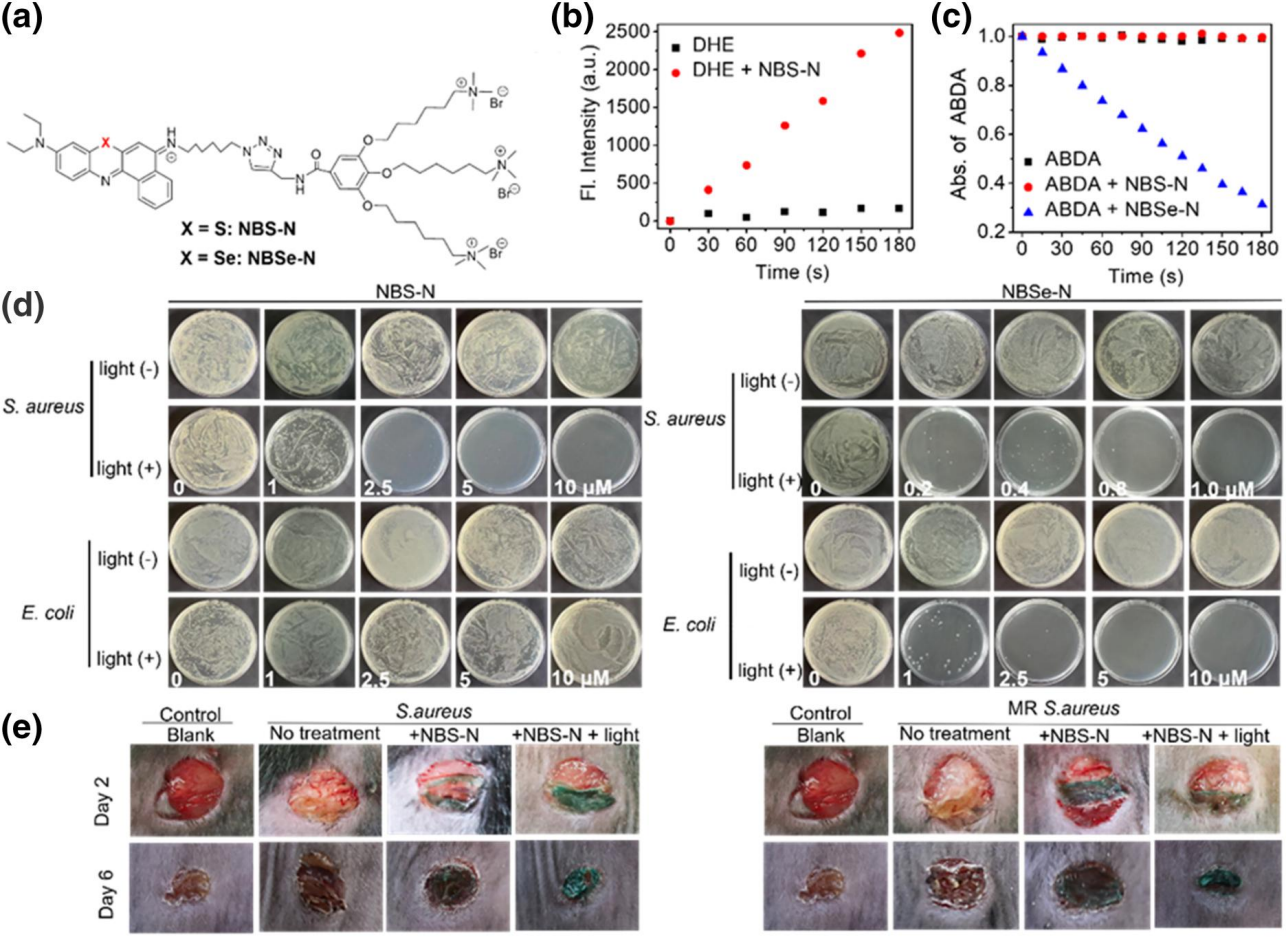
(a) Chemical structures of **NBS‐N** and **NBSe‐N**. (b) O_2_^•−^ generation capability of **NBS‐N**. (c) Comparison of ^1^O_2_ generation yields of **NBS‐N** and **NBSe‐N**. (d) Antibacterial tests of **NBS‐N** and **NBSe‐N** against *Staphylococcus aureus* and *Escherichia coli*. (e) Photographs of *S. aureus*‐ and methicillin‐resistant *S. aureus*‐infected wounds in mice treated with or without **NBS‐N** in the absence or presence of red‐light irradiation on Day 2 and Day 6. Reproduced with permission from Ref. [34]. Copyright (2022) Wiley‐VCH.

In certain cases, the selective elimination of bacteria is essential for maintaining a balanced microbial environment. However, engineering a selective agent to kill gram‐positive bacteria is a challenging task. As depicted in Figure 13, a proof‐of‐concept study established that a red‐light‐absorbing photodynamic agent (**NBS‐N**) that primarily generates superoxide anion radicals (O_2_^•−^) can selectively kill gram‐positive bacteria. By contrast, a photodynamic agent (**NBSe‐N**) with a higher singlet oxygen (^1^O_2_) yield can eliminate both gram‐positive and gram‐negative bacteria.^[34]^ **NBS‐N** was prepared based on the phenothiazinium structure with three cationic groups in order to bind to the negative charges of the bacterial membrane, whereas **NBSe‐N** was synthesized by substituting selenium for sulfur in **NBS‐N**. These experiments demonstrated in vitro bacterial fluorescence imaging and morphological changes as well as in vivo treatment of gram‐positive bacteria‐infected mouse skin, presumably providing a clue for developing photodynamic agents that rely on various ROS to inhibit bacterial activity.

**FIGURE 14:**
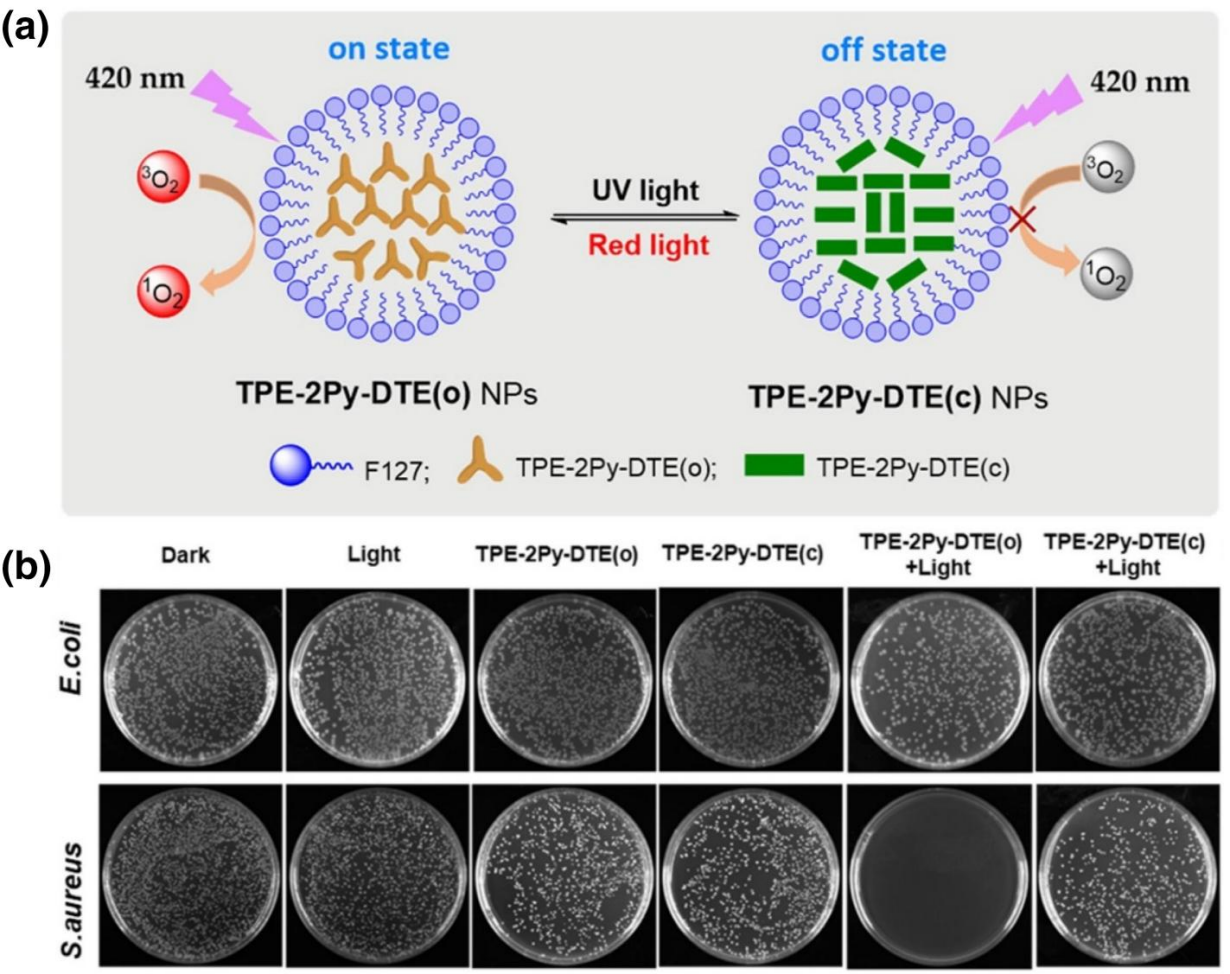
(a) Structure of **TPE‐2Py‐DTE(o)** and **TPE‐2Py‐DTE(c)** nanoparticle and its photoswitching behavior. (b) Different antibacterial effects of **TPE‐2Py‐DTE(o)** and **TPE‐2Py‐DTE(c)** in *Staphylococcus aureus*‐ and *Escherichia coli.* Reproduced from Ref. [35]. Copyright (2022) with permission from Elsevier.

Similar to the case of NBS‐N above, a **TPE‐2Py‐DTE** nanoparticle capable of selective eradication of Gram‐positive bacteria was designed.^[35]^ Using Py‐TPE as an AIE‐based fragment and DTE as a switch unit, **TPE‐2Py‐DTE** exhibits both AIE characteristics and photoswitching behavior. When irradiated with 365 nm UV light under organic solvent conditions, **TPE‐2Py‐DTE** changes from the open to the closed form, and the color of the emitted light changes from yellow to green; upon irradiation with red light (*λ* = 620–630 nm, 40 s), it returns to the yellow state. ^1^O2 generation efficiency of **TPE‐2Py‐DTE(o) NPs** is about 5.6 times higher than that of **TPE‐2Py‐DTE(c) NPs**, indicating excellent light control capability. As a result, **TPE‐2Py‐DTE(o)** was more effective in killing gram‐positive bacteria compared to **TPE‐2Py‐DTE(c)** and the breakdown of bacterial cell walls was additionally observed (Figure 14).

## 3 CONCLUSIONS

Because of the shortcomings of the existing antibiotics, treatment of bacterial infection by PDT has come under the spotlight, and further comprehensive studies on the realization of on‐demand activation of PDT with phototoxicity only in the treatment process are required. Antimicrobial PDT and PTT are emerging as effective treatments for bacterial infection, especially for MDR bacterial infections. ROS and heat produced by PSs for PDT and PTT, respectively, are effective for antibacterial therapies. The recently demonstrated SODS allows us to control the different photophysical properties of PSs for PDT, PTT, PA imaging, fluorescent imaging, and so on. In addition, understanding the differences between the membranes of gram‐negative and gram‐positive bacteria along with the SODS enable more efficient and selective antimicrobial PDT and PTT.

We reported the nano assembly of amphiphilic phthlocyanine‐based type I PSs, which can show an effective antibacterial efficacy. On the other hand, the phthalocyanine‐derivative bearing 3‐(dimethylamino) phenoxy group forms NanoPcN, which produces ROS via ISC and heat via vibration relaxation. Interestingly, in the case of PcN4‐BA, which is a self‐assembled nanostructure of a phthalocyanine derivative with quaternary ammonium and BA, the amount of ROS produced gradually increases because of the active ISC in the aggregated molecules. The conjugates of spermine molecules with the porphyrin, PN3, were self‐assembled into a pH‐sensitive nanoporphyrin (PN3‐NP), which was used for NIR fluorescence imaging and photoablation of biofilms. In the AIEgens‐based PSs, the phenylboronic acid moiety is linked to the AIEgens such that the BDPTV AIEgen can become tightly attached to the bacterial cell wall. BDPTV showed an excellent selectivity for killing gram‐positive bacteria, such as *S. Aureus* and *MRSA*. On the other hand, a rhodamine‐based AIE PS, **CS‐2I**, can also selectively stain and kill gram‐positive bacteria under photoirradiation. Finally, a proof‐of‐concept study revealed that **NBS‐N**, which primarily generates superoxide anion radicals (O_2_^•−^), can selectively kill gram‐positive bacteria. In contrast, **NBSe‐N**, which exhibits a higher singlet oxygen (^1^O_2_) yield, is suitable for eliminating both gram‐positive and gram‐negative bacteria.

We believe that investigations on PSs for bacterial infection treatment via PDT and PTT will continue in the near future to provide better treatment and resistance against bacterial infectious diseases. This mini review highlights that the recently developed SODS can be a very powerful tool to achieve the aforementioned goals.

## CONFLICT OF INTEREST STATEMENT

The authors declare no conflicts of interest.
